# Callus induction and regeneration via shoot tips of *Dendrocalamus hamiltonii*

**DOI:** 10.1186/s40064-016-3520-7

**Published:** 2016-10-18

**Authors:** Qiaolu Zang, Ling Zhou, Fei Zhuge, Haiyun Yang, Xiaoqin Wang, Xinchun Lin

**Affiliations:** The Research Center for the State Key Laboratory of Subtropical Silviculture, Zhejiang Agriculture and Forestry University, 88 North Circular Road, Lin’an, 311300 Zhejiang People’s Republic of China

**Keywords:** Callus induction, *Dendrocalamus hamiltonii*, Regeneration, Shoot tips

## Abstract

By using shoot tips as explants, various media and culture conditions for callus induction and proliferation, shoot differentiation, root induction and plantlet transplantation to develop an efficient and reliable regeneration system with *Dendrocalamus hamiltonii* were tested. Murashige and Skoog (MS) medium supplemented with 3 mg/l 2, 4-dichlorophenoxyacetic acid, 1 mg/l benzyladenine (BA), 500 mg/l glutamine, 500 mg/l proline, and 500 mg/l casein hydrolysate yielded the best rates of callus induction and granular-compact callus induction. MS medium supplemented with 1 mg/l BA, 0.3 mg/l kinetin and 0.3 mg/l naphthaleneacetic acid conferred the highest differentiation rate of calli. The maximum rooting rate was obtained in 1/2 MS medium supplemented with 3 mg/l indole-3-butyric acid, and the roots were long and thick. All hardened plantlets survived after transfer to an equal ratio mixture of peat, vermiculite and perlite. The regeneration system of *D. hamiltonii* developed is efficient and provides a useful tool for genetic transformation in bamboo species.

## Background

Bamboos are the members of the grass family, including more than 88 genera and 1400 species worldwide. Because of rapid growth, high output, highly maintaining soil and water, and other values, they are economically, socially and ecologically important in China (Jiang 2002). *Dendrocalamus hamiltonii* is one of the three most important bamboo species with sweet shoots in the world and is cultivated for its shoots and timber in Xishuangbanna and Puer, Yunnan province, in China.

Regeneration from calli is a useful approach for the genetic improvement of bamboo. Regenerated plantlets in bamboo were obtained for the first time from the zygotic embryos of *Bambusa arundinacea* (Mehta et al. 1982). Then the mature seeds of *D. strictus*, *D. latiflorus*, *Bambusa multiplex* and *D. hamiltonii* (Rao et al. 1985; Yeh and Chang 1987; Yuan et al. 2009; Zhang et al. 2010), and young inflorescences of *B. oldhami*, *B. beecheyana* var. *beecheyana* and *D. latiflorus* (Yeh and Chang 1986a, b; Qiao et al. 2013) were used as explants for callus induction and regeneration.

The inflorescences, embryos and seeds of the bamboos are good resources for explants, but they are difficult to obtain because bamboos rarely blossom and bear fruit. However, shoot tips are available at any time and easy to obtain. Thus, shoot tips can be used as explants to create regenerated plantlets through callus induction and regeneration.

In this study, the effects of different media and different combinations of 2, 4-dichlorophenoxyacetic acid (2, 4-D), benzyladenine (BA), indole-3-butyricacid (IBA), and organic additives on callus induction from shoot tips of *D. hamiltonii* were determined. The orthogonal test design [L9 (3^4^)] was used to investigate the effects of BA, α-naphthaleneacetic acid (NAA), and kinetin (KT) on shoot differentiation. An efficient regeneration system was developed that can be used for genetic improvement of bamboo.

## Methods

### Plant materials and tissue culture condition

**Fig. 1:**
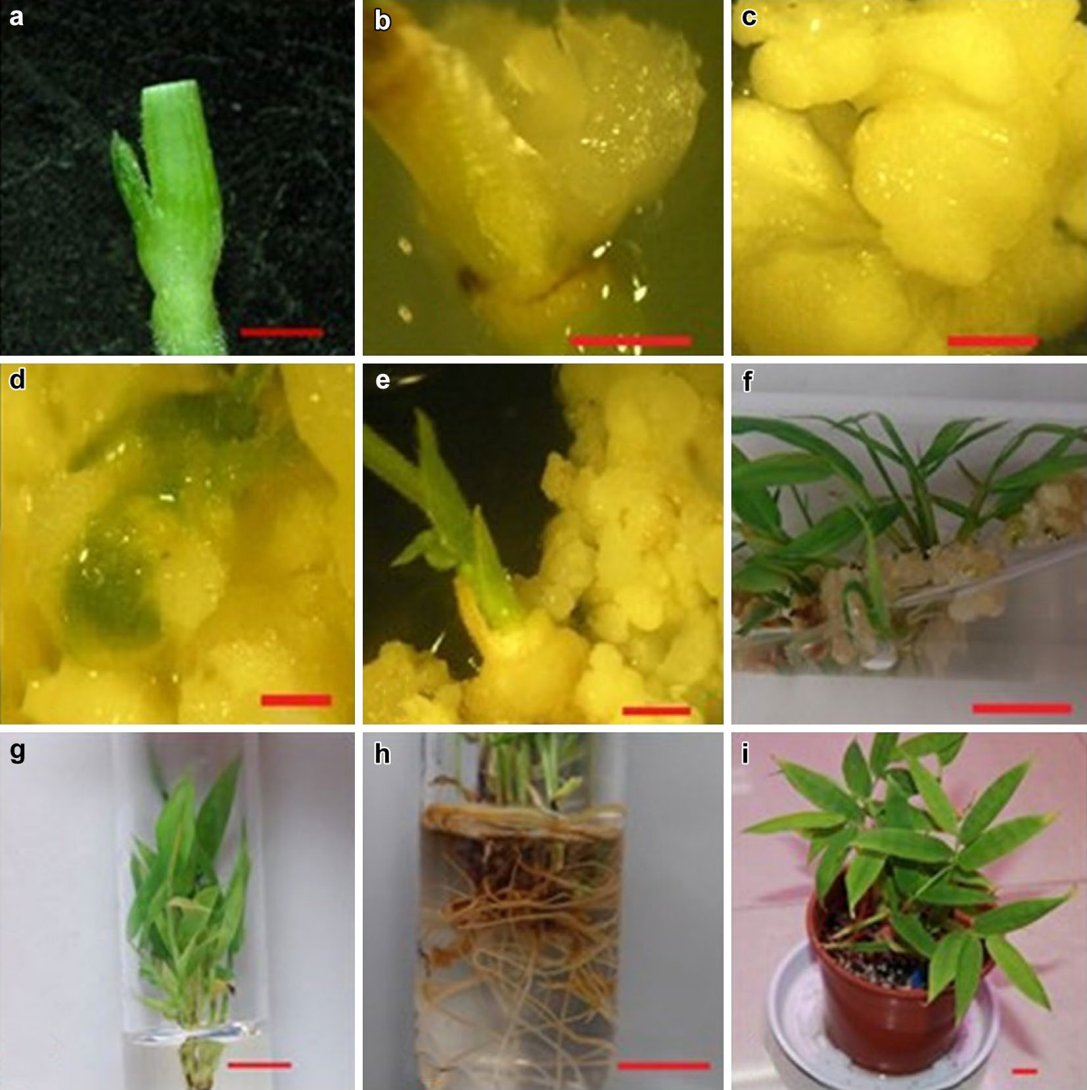
Plantlets regenerated from shoot tips of *D. hamiltonii.* **a** shoot-tips; **b** callus formation from shoot-tips; **c** granular and compact calli; **d** calli turning green; **e**, **f** buds protruding from calli; **g** plantlets developed in cluster; **h** root induction of shoots; **i** plantlets after transferred to soil pot. **a–e** *bar* 10 µm; **f–i** *bar* 100 µm

*Dendrocalamus hamiltonii* (Poaceae, subfamily bambusoideae) was obtained from Xishuangbanna, Yunnan Province, P. R. China. Shoots were collected from the plants cultivated in greenhouse of Zhejiang Agriculture and Forestry University in August. For surface disinfection, the shoots were first washed with running tap water for about 1 h, then immersed in 75 % (v/v) ethanol for 30 s, followed by 1 % (v/v) sodium hypochloride (NaClO) vacuum infiltration for 15 min. The shoots were separated and the shoot-tips were inoculated on tissue culture media (Fig. 1a) after the shoots were washed with autoclaved distilled water six times. The callus induction and subculture were conducted in the dark at 25 ± 2 °C, and the shoot differentiation induction and root induction were performed under a 16/8-h light/dark photoperiod with continuous illumination of 2400 lx at 25 ± 2 °C.

### Callus induction and subculture

Shoot-tips were incubated on various basal media, including MS medium (Murashige and Skoog 1962), half-strength MS macrosalts (1/2 MS), B5 (Gamborg et al. 1968), Nitsch (1951) and White (1943).

The different concentrations of 2, 4-D (0, 0.1, 0.3, 1, 3 and 10 mg/l), BA (0, 1, 2 and 4 mg/l), organic additives [500 mg/l casein hydrolysate (CH), 500 mg/l proline (Pro), 500 mg/l glutamine (Gln), 30 mg/l adenine sulfate (Ads), 500 mg/l yeast extract (YE), and 500 mg/l CH + 500 mg/l Pro + 500 mg/l Gln)], 30 g/l sucrose and 8 g/l Type A agar were used to select the optimal medium for callus induction. The pH of medium was adjusted to 5.7. Compact and granular calli were subcultured on a new medium every 4 weeks.

### Adventitious shoot differentiation induction

After 2–3 subcultures, creamy-yellow and compact calli were transferred to MS medium supplemented with 30 g/l sucrose, 3 g/l gelrite and different concentrations of plant growth regulators (PGRs), including BA (1, 2 and 4 mg/l), KT (0.1, 0.3 and 1 mg/l) and NAA (0.1, 0.3 and 1 mg/l) in an orthogonal array [L_9_ (3^4^)], which is a time- and cost-saving strategy to investigate the main effects in order of priority (Ross 1996; Rao et al. 2008). The pH of the medium was adjusted to 5.7. The differentiation rate and shoot growth condition were recorded 4 weeks later.

### Rooting and transplantation

After 4 weeks’ differentiation, the differentiated shoots (length 3 cm) were induced on the 1/2 MS medium supplemented with 30 g/l sucrose, 3 g/l gelrite and IBA in different concentrations (0, 1, 3 and 10 mg/l) for 4 weeks. Plantlets that rooted well were transferred to a cultivation chamber (20000 lx) for hardening in the greenhouse. The survival rate was recorded after 4 weeks.

### Statistical analysis

All treatments were repeated three times. ANOVA involved use of SPSS v15 (SPSS Inc., Chicago, IL). The differences between treatments were evaluated by Duncan’s multiple range test (Duncan 1955). Figures were created by using SigmaPlot v8.0, and DPS v6.55 was used to analyze orthogonal test.

## Results

### Effect of basal media and plant growth regulators on callus induction

**Fig. 2:**
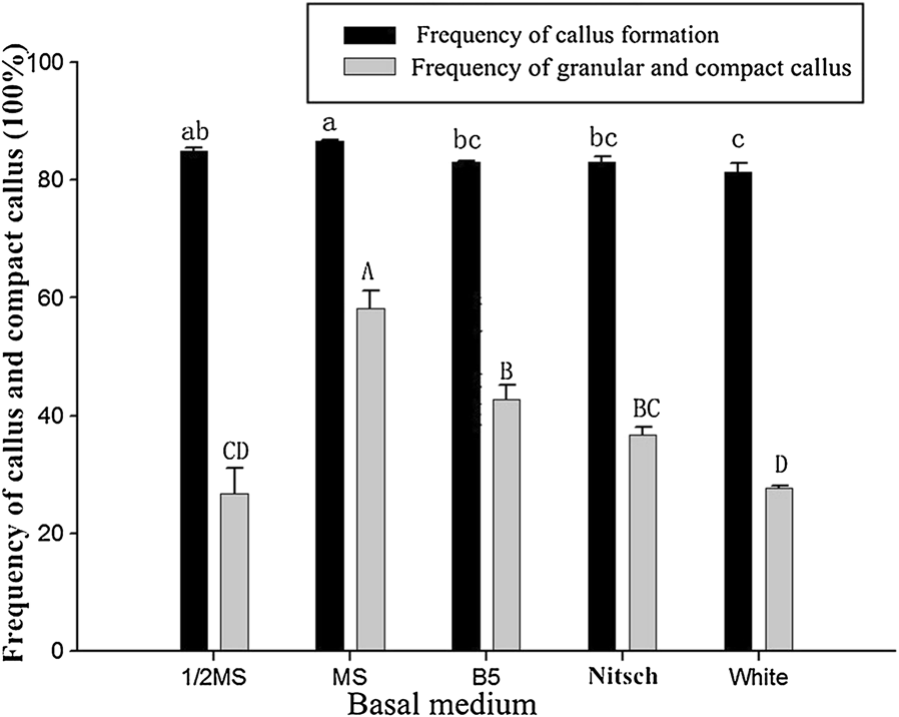
Effect of different basal media on callus and compact callus induction from shoot-tips of *D. hamiltonii*. Basal media of MS, half-strength MS macrosalts (1/2 MS), B5, Nitsch and White were tested for callus induction. Three replications of 20 explants each were used. Values within the *same column* followed by the *same letter* are not significantly according to the least significant difference at *P* *<* 0.05 (Duncan 1955). All data were collected 4 weeks later

Calli appeared from the shoot tips on the media supplemented with 3 mg/l 2, 4-D at about 2 weeks after inoculation (Fig. 1b) and grew well 1 month later. The rate of callus induction on MS medium was 94.60 % and significantly higher than that on B5, Nitsch and White media but did not differ from that on 1/2 MS medium (Fig. 2). Most of the calli grown on 1/2 MS medium were loose lumps with long buds or had a fibrous surface and were unable to proliferate and differentiate, whereas most (58.27 %) induced on MS medium were compact and granular and were able to proliferate (Fig. 1c, d). MS medium was the best for callus induction from shoot tips of *D. hamiltonii*.

**Fig. 3:**
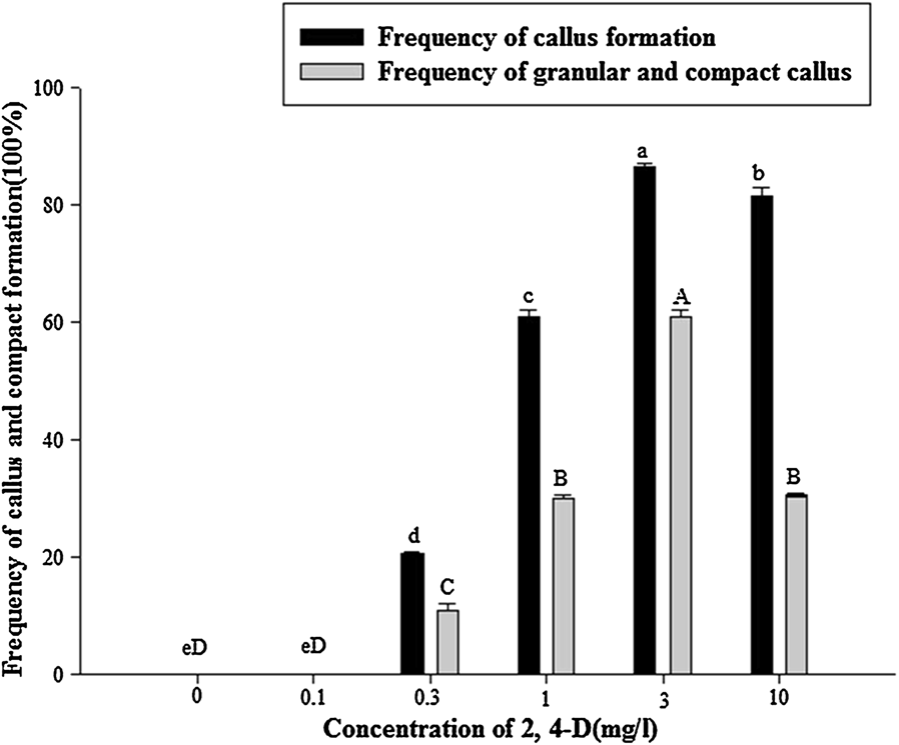
Effect of 2, 4-D on callus and compact callus induction from shoot-tips of *D. hamiltonii*. The explants were cultured on the MS medium supplemented with 30 g/l sucrose, and 8 g/l Type A agar, and treated with the different concentrations of 2, 4-D (0, 0.1, 0.3, 1, 3 and 10 mg/l). Three replications of 20 explants each were used. Values within the *same column* followed by the *same letter* are not significantly different according to the least significant difference at P < 0.05 (Duncan 1955). All data were collected 4 weeks after treatment with 2, 4-D

2, 4-D is crucial for callus induction, but different concentrations may be required for different explants. Therefore, the effects of the different concentrations of 2, 4-D on callus formation were determined. The rates of callus induction (86.50 %) and compact and granular callus (61.00 %) were all elevated with increasing 2,4-D concentrations but were decreased with 2,4-D at 10 mg/l (Fig. 3). The data suggest that 3 mg/l was the optimal 2, 4-D concentration for callus induction.

**Fig. 4:**
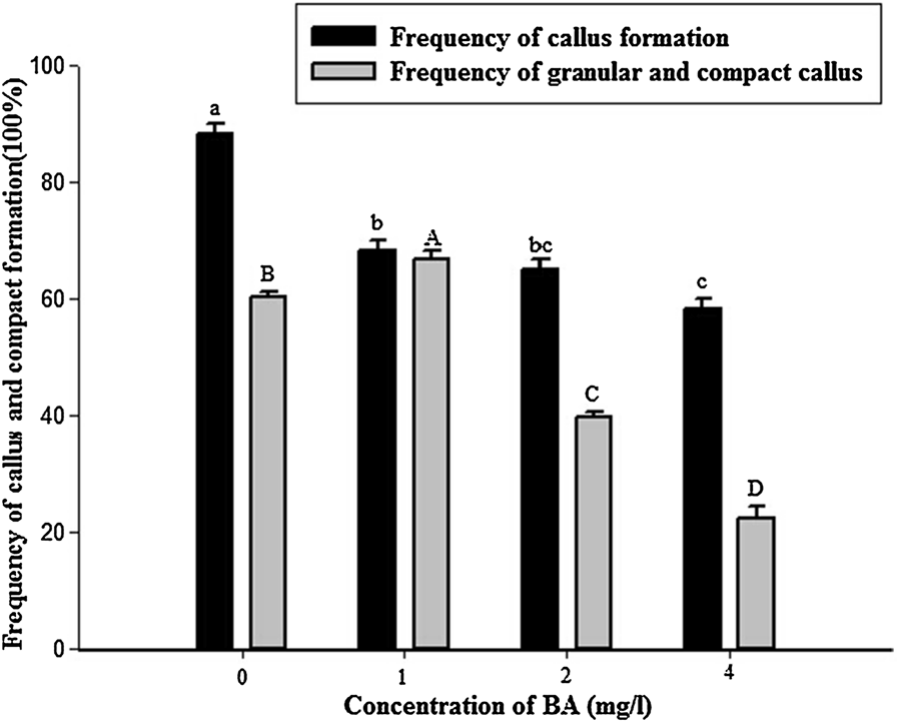
Effect of the different concentrations of BA on callus and compact callus induction from shoot-tips of *D. hamiltonii*. The explants were cultured on the MS medium supplemented with 3 mg/l 2, 4-D, 30 g/l sucrose, and 8 g/l Type A agar, and treated with the different concentrations of BA (0, 1, 2 and 4 mg/l). Three replications of 20 explants each were used. Values within the *same column* followed by the *same letter* are not significantly different according to the least significant difference at *P* < 0.05 (Duncan 1955). All data were collected 4 weeks later

Different concentrations of BA combined with 3 mg/l 2, 4-D were used to determine the effect of BA on callus formation. The rate of callus induction was higher without BA (88.35 %) than with any BA concentrations tested (58.35–68.35 %), but the rate of compact and granular calli was higher with 1 mg/l BA (66.94 %) than with the other treatments (22.29–60.50 %) (Fig. 4). Therefore, 1 mg/l BA was the best for producing high-quality calli.

**Fig. 5:**
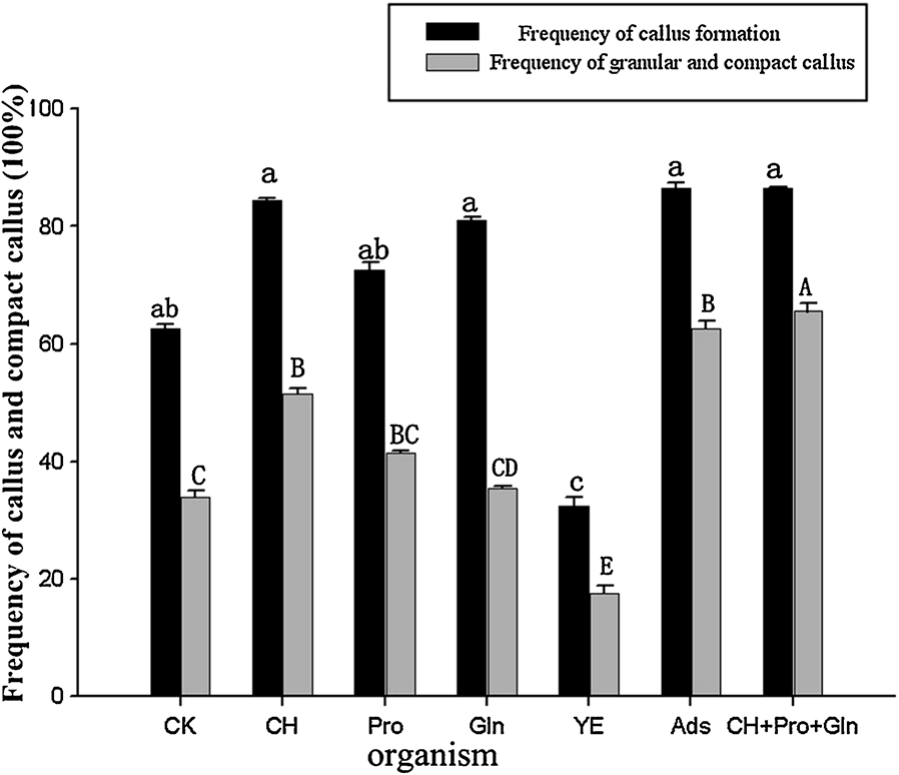
Effect of organic addictives on callus and compact callus induction from shoot-tips of *D. hamiltonii.* The explants were cultured on the MS medium supplemented with 3 mg/l 2, 4-D, 1 mg/l BA, 30 g/l sucrose, and 8 g/l Type A agar, and treated with different organic addictives (500 mg/l CH, 500 mg/l Pro, 500 mg/l Gln, 30 mg/l Ads, 500 mg/l YE, and 500 mg/l CH + 500 mg/l Pro + 500 mg/l Gln). Three replications of 20 explants each were used. Values within the *same column* followed by the *same letter* are not significantly different according to the least significant difference at *P* < 0.05 (Duncan 1955). All data were collected 4 weeks later

The effects of different organic additives on callus formation were also determined. Organic additives except yeast extraction increased the rate of callus induction (70.00–86.70 %) as compared with the control (61.67 %) (Fig. 5). As well, the combination of CH, Pro and Gln yielded the highest rate of compact callus induction (63.30 %), which significantly differed from that with other treatments (16.67–60.00 %).

### Effect of plant growth regulators on shoot differentiation induction

The effect of PGRs on shoot differentiation and development was shown in Table 1. The combination of 1 mg/l BA, 0.3 mg/l KT and 0.3 mg/l NAA yielded better-quality shoots and the highest rate of shoot differentiation (51.65 %) than other treatments (Fig. 1d–g). The effect of individual factors on shoot differentiation rate decreased in the order of NAA > KT > BA but not significantly (Table 2).

**Table 1:** Effect of plant growth regulators (PGRs) on shoot differentiation of *D. hamiltonii*

Orthogonal combination	PGRs (mg/l)			Shoot differentiation rate (%)	Shoot growth pattern
	BA	KT	NAA		
1	1	0.1	0.1	21.67 ± 1.67^c^	Yellow–green; few buds
2	1	0.3	0.3	51.65 ± 1.65^a^	Yellow–green; fascicled buds
3	1	1	1	18.39 ± 1.62^d^	Yellow–green; light browning
4	2	0.1	0.3	28.35 ± 1.65^b^	Yellow–green; few buds
5	2	0.3	1	23.32 ± 0.02^bc^	Yellow–green; few buds
6	2	1	0.1	21.67 ± 1.67^c^	Yellow–green; few buds
7	4	0.1	1	18.39 ± 1.62^d^	Yellow–green; few buds
8	4	0.3	0.1	21.67 ± 1.67^c^	Yellow–green; few buds
9	4	1	0.3	20.00 ± 0.02^c,d^	Yellow–green; albino
X1	30.57	22.80	21.67		
X2	24.45	32.21	33.33		
X3	20.02	20.02	20.03		
R	10.55	12.19	13.30		

Values represent the mean ± standard error. Values within the same column followed by the same letter are not significantly different according to the least significant difference at P < 0.05 (Duncan 1955)

X mean value of the test corresponding to a factor in the same level. R = Xmax − Xmin

Each treatment consisted of three replications of 20 explants each. All data were collected 4 weeks after inoculated into the medium supplemented with PGRs

**Table 2:** ANOVA of effect of PGRs on the shoot differentiation rate of *D*. *hamiltonii*

Sources of variance	Sum of squares	Degrees of freedom	F value	F(0.05)
BA	168.39	2	1.20	19.00
KT	244.97	2	1.74	19.00
NAA	315.60	2	2.24	19.00
e	140.95	2		

*e* Sum of squares of experimental variance

### Rooting and transplantation

The rate of root induction and number of roots were both increased with increasing IBA concentration (Table 3). However, 10 mg/l IBA caused swollen roots, and roots induced by 3 mg/l IBA were stout (Fig. 1h). These data demonstrate that 3 mg/l IBA was the optimal concentration for rooting induction on 1/2 MS medium.

**Table 3:** Effect of different concentrations of IBA on root induction of *D. hamiltonii*

IBA (mg/l)	Rate of root induction (%)	Number of roots	Root status
0	40	4.00 ± 0.58^a^	Slender
1	65	3.67 ± 0.33^a^	Slender
3	95	7.67 ± 0.88^b^	Stout
10	90	7.33 ± 1.30^b^	Swollen

Values represent the mean ± standard error. Values within the same column followed by the same letter are not significantly different according to the least significant difference at P < 0.05 (Duncan 1955)

Each treatment consisted of three replications of 20 explants each. All data were collected 4 weeks after inoculated into the medium supplemented with IBA

The survival rate of hardening plantlets was 100 % after they were transferred to an equal ratio mixture of peat, vermiculite and perlite and grew well in the greenhouse (Fig. 1i).

## Discussion

Genetically improving bamboo species by using traditional sexual crossbreeding is difficult because bamboo takes many years to blossom and then dies. A transgenic approach provides a useful tool for genetically breeding bamboo. A stable and efficient regeneration system is important for genetic improvement. A regeneration system involving *D. hamiltonii* with zygotic embryos in mature seeds, which are difficult to obtain, was developed previously (Zhang et al. 2010). In this study, shoot tips that are available all the time were used as explants, which made the regeneration system be more convenient and feasible.

Basal medium is an important factor to induce callus formation in plants. Different species may require different media for callus induction. For instance, B5 medium was used for culture of legumes and woody plants that are sensitive to ammonium salt poisoning (Ma and Zhang 2007). The regeneration of bamboos via explants such as node segments (Godbole et al. 2002) and seeds (Woods et al. 1992) required MS and B5 media, respectively. Moreover, Nitsch basal medium was also used to induce somatic embryogenesis (Martinelli et al. 2015; Morgana et al. 2015). In this paper, MS, B5, Nitsch, White and 1/2 MS media were tested for calli formation, and MS was the best basal medium for the regeneration system in *D. hamiltonii*.

2, 4-D was essential to callus induction in bamboo, but a high concentration reduced the capacity of calli to differentiate (Huang et al. 1989; Enric et al. 2000). In bamboo, an optimal concentration of 2, 4-D depends on the species (Saxena and Dhawan 1999; Lin et al. 2003; Woods et al. 1992). In this study, 3 mg/l 2, 4-D was the best concentration to induce calli, which were compact and granular and proliferate rapidly.

The appropriate concentration of BA promoted callus induction and differentiation in many plant species (Liu et al. 2009; Liu and Shi 1996). In addition, the combination of 2,4-D and BA was efficient in inducing compact callus and plant regeneration of many species (Wang et al. 2007; Siddique and Islam 2015). In this study, BA played a role in improving bamboo callus induction and proliferation.

Organic additives were used to increase nutrition sources and improve the quality of calli in tissue culture (Staden and Drewes 1975; Dix and Staden 1982; Qiao et al. 2013). Coconut milk, honey, banana extract, yeast extract, malt extract, hydrolyzed casein, and various amino acids individually or in combination played a significant role in reducing callus browning, adjusting osmosis, and producing secondary (Kim et al. 2009; Indrayanto et al. 1995; Armstrong and Green 1984). In this paper, the combination of 500 mg/l CH, 500 mg/l Pro and 500 mg/l Gln greatly promoted callus induction in *D. hamiltonii*.

The stage of callus differentiation is a key to establishing a regeneration system. Organ differentiation was mainly determined by the balance of hormones, which is changed by adjusting appropriate exogenous cytokinin and auxin levels (Shirin and Rana 2007). A high concentration of BA might cause plant growth inhibition or have toxic effects on bamboo growth. A low concentration of KT played an active role in differentiation, whereas a high concentration could cause bud browning and unhealthy plants (Nadgir et al. 1984; Chambers et al. 1991). In the study, calli subcultured in MS medium supplemented with 1 mg/l BA, 0.3 mg/l KT, and 0.3 mg/l NAA produced fascicled shoots that showed vigorous budding and elongation.

A medium supplemented with a certain concentration of an auxin was found conducive to bamboo rooting (Huang and Murashige 1983). In this paper, with 3 mg/l IBA, the rate of root induction peaked (95 %), and each plantlet generated 7–8 roots that grew well with the supplement.

## Conclusion

A stable and efficient regeneration system in *D. hamiltonii* was developed by using easily obtained shoot tips as explants, which provides a useful tool for genetic transformation in bamboo species.

## Abbreviations

MS medium: Murashige and Skoog medium
2, 4-D: 2, 4-dichlorophenoxyacetic acid
NAA: α-naphthaleneacetic acid
BA: 6-benzyladenine
Gln: glutamine
Pro: proline
CH: casein hydrolysate
Ads: adenine sulfate
PGRs: plant growth regulators
KT: kinetin
IBA: indole-3-butyric acid
